# Therapeutic enzyme engineering using a generative neural network

**DOI:** 10.1038/s41598-022-05195-x

**Published:** 2022-01-27

**Authors:** Andrew Giessel, Athanasios Dousis, Kanchana Ravichandran, Kevin Smith, Sreyoshi Sur, Iain McFadyen, Wei Zheng, Stuart Licht

**Affiliations:** Moderna Therapeutics, 200 Technology Square, Cambridge, MA 02139 USA

**Keywords:** Machine learning, Protein design

## Abstract

Enhancing the potency of mRNA therapeutics is an important objective for treating rare diseases, since it may enable lower and less-frequent dosing. Enzyme engineering can increase potency of mRNA therapeutics by improving the expression, half-life, and catalytic efficiency of the mRNA-encoded enzymes. However, sequence space is incomprehensibly vast, and methods to map sequence to function (computationally or experimentally) are inaccurate or time-/labor-intensive. Here, we present a novel, broadly applicable engineering method that combines deep latent variable modelling of sequence co-evolution with automated protein library design and construction to rapidly identify metabolic enzyme variants that are both more thermally stable and more catalytically active. We apply this approach to improve the potency of ornithine transcarbamylase (OTC), a urea cycle enzyme for which loss of catalytic activity causes a rare but serious metabolic disease.

## Introduction

Protein engineering has the potential to improve on natural enzymes by altering their functional properties, including stability, catalytic activity, and substrate specificity^1^. Engineering strategies such as rational design and directed evolution have had remarkable success in applications ranging from industrial chemical synthesis and biodegradation to biosensing^2–6^. Therapeutic enzymes may also benefit from such efforts: more stable or catalytically active proteins would have the potential to enable decreased dosages or dosing frequency, thereby reducing manufacturing costs and facilitating patient compliance by simplifying therapeutic regimens. However, therapeutic proteins must function in vivo, and thus the number of mutations in a therapeutic enzyme should ideally be kept to a minimum to reduce potential immunogenicity^6^.

Amino acid sequence alignments of protein families are powerful tools for exploring functional sequence space and understanding the relationships between sequence and function. Protein engineering methods that leverage sequence conservation, such as consensus sequence design, have demonstrated success in improving protein stability, but these methods require screening of many sequence variants over several rounds^7–9^. Models of pairwise sequence covariance, such as Statistical Coupling Analysis (SCA)^10–13^, add another layer of evolutionary detail for protein engineering^11–14^, but they still fall short of capturing the full and complex epistatic relationships that exist between locations distributed across the entire protein sequence. It has been estimated that while a typical site in a protein can tolerate an average of 8 natural amino acid types, about 90 percent of all amino acid substitutions are neutral or beneficial only in the context of their genetic background^15^. Thus, the most effective sequence design methods may need to consider not just position- or pair-wise conservation when making mutations, but also the interactions between all positions in a given sequence alignment.

Despite the potential importance of utilizing all the positional information in a set of functional protein sequences, extracting this information in useful form is not straightforward. However, the ever-increasing number of sequenced genomes and advances in machine learning have created new possibilities for predicting (and maximizing) protein function from sequence^16^. One approach has been to build regression models from mutation datasets, which enables either interpretation of the parameters of these models to guide manual sequence design or combination of the models with search heuristics to optimize over sequences directly. Recent work has improved this methodology by using data-driven numerical representations of sequences as input^17,18^, deploying more powerful models^18–21^, and/or applying more effective optimization techniques^22–26^. An alternative approach has been to directly model the distributions of families of protein sequences using generative deep learning^18,24,27–34^. This approach allows for sampling novel sequences, but most studies have not tested novel variants in vitro or in vivo.

The advent of mRNA therapeutics and gene editing technologies expands the scope of therapeutic protein engineering beyond extracellular proteins to intracellular proteins as well. An example of an intracellular therapeutic target that might benefit from protein engineering is ornithine transcarbamylase (OTC), a metabolic enzyme that, in eukaryotes, is localized in the mitochondrion and functions in the urea cycle, and, in prokaryotes, functions in arginine biosynthesis^35,36^. OTC deficiency is the most common urea cycle disorder in humans, affecting approximately 1 in 60,000 to 1 in 72,000 births^37^. Liver transplantation may be curative but requires assessment on a case-by-case basis. Human OTC (hOTC) has at least 312 disease-causing mutations, and non-synonymous mutations are found at 150 unique locations across the entire length of the protein^37^.

OTC's role in arginine biosynthesis has resulted in a high degree of functional conservation with high sequence diversity across all kingdoms of life. For example, the *E. coli* variants ArgF and ArgI have 5–10 × higher specific activity^38^, but only 33% sequence identity with hOTC. This combination of functional conservation and sequence diversity suggests an extensive landscape of functional sequence space to draw upon to engineer improved catalytic activity, stability, or both.

In this work, we apply these recent advances in generative deep learning to the problem of engineering an improved OTC. By training a neural network on a large alignment of OTC sequences, we sample novel, near-human versions of OTC that maintain the correlations present in known functional OTCs. The majority of these novel variants exhibit improved stability, specific activity, or both. The deep learning derived library outperforms a consensus library that does not incorporate residue-residue correlations, suggesting that these correlations make a meaningful contribution to enzyme function in this system. Taken together, these results also suggest that, by virtue of its ability to capture the diversity of existing sequences directly, generative modeling has great potential utility for engineering proteins with improved functional properties.

## Results

### Neural network modeling and library design

**Figure 1:**
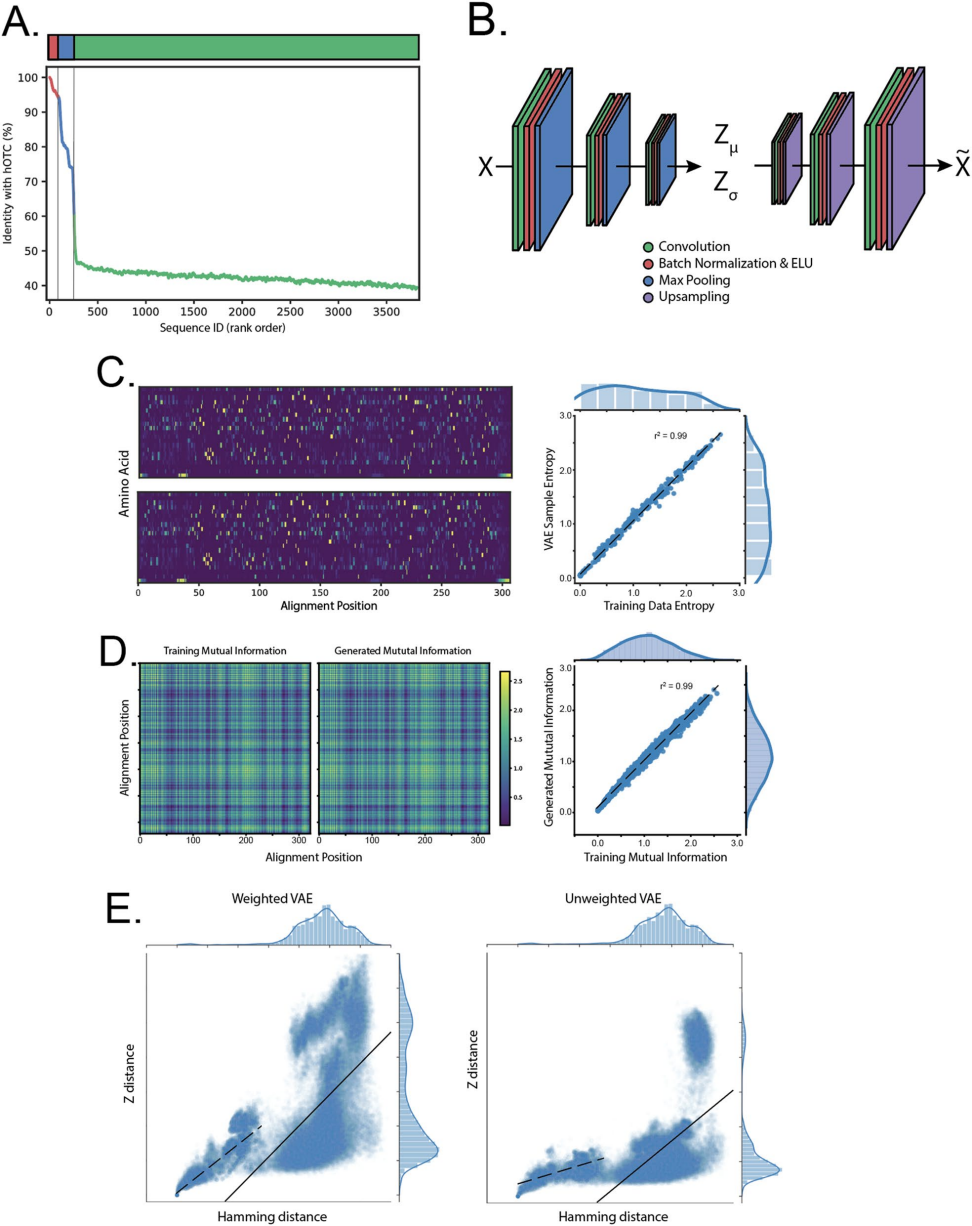
A Variational Autoencoder (VAE) effectively models co-evolutionary dependencies in ornithine transcarbamylase (OTC). (**A**) Species distribution of the training dataset. Top, relative percentages of mammalian, eukaryotic and prokaryotic sequences, ordered by Hamming distance to human OTC (hOTC). Bottom, Hamming distances to hOTC for all sequences used in the training data. (**B**) VAE model schematic and parameters. The VAE consists of an encoding convolutional neural network (CNN) (left) which takes one-hot encoded sequences as input, and outputs the parameters of a multivariate Gaussian distribution (${\mathrm{Z}}_{\upmu }$ and ${\mathrm{Z}}_{\upsigma }$). Samples from this distribution ($\mathrm{Z}$) are passed through a second decoding CNN. Both the encoder and decoder consist of multiple layers of convolution, batch normalization and pooling/up-sampling. The model is trained end-to-end to reproduce its input and regularized such that the distribution $\mathrm{Z}$ matches a Gaussian prior. (**C**) Per position amino acid frequency in VAE samples match those in the training data. Top left, average training data. Columns are distributions over amino acid frequency at each location in the alignment. Bottom left, as above, but for samples from the VAE model. Right, scatter plot of all amino acid frequencies (training vs. samples), (Pearson's ${\mathrm{R}}^{2}$ value of 0.99). (**D**) Second-order correlations in VAE samples match those in the training data. Left, mutual information between all alignment locations in the training data. Middle, mutual information between all alignment location in VAE samples. Right, a scatter plot of position-to-position mutual information between the training data and the samples (Pearson's ${\mathrm{R}}^{2}$ value of 0.99). (**E**) Positive correlation between Hamming and latent space distance. Left, scatter plot of Hamming distance and Euclidean distance in Z for a model with sequences weighted proportional to their Hamming distance to hOTC; right, same but with all sequences weighted equally. Overall correlations when considering all sequences are similar (Pearson's ${\mathrm{R}}^{2}$ values of 0.25 vs 0.26, solid lines). In the weighted model, the 5% most similar training data exhibit higher correlation than the training data as a whole (Pearson's ${\mathrm{R}}^{2}$ values of 0.81 vs 0.56, dashed lines).

To leverage evolutionary information to improve OTC activity, we built a large multi-sequence alignment of OTC homologs obtained by BLAST search. After filtering these sequences to remove those that caused the most gaps, we were left with 3818 sequences, of which approximately 100 were mammalian, 263 were eukaryotic or archaea, and the remaining 3558 were prokaryotic (Fig. 1A). These sequences formed the basis of our training data set and spanned a range of similarities, with microbial sequences averaging about 45% similarity with each other and 58% similarity with hOTC.

To better capture the full extent of co-evolutionary conservation present in known OTC sequences, we applied variational autoencoders (VAEs), a recent advance from the deep learning literature^39^. VAEs are probabilistic neural network models comprising of an encoder, a stochastic sampling step, and a decoder (Fig. 1B). The encoder maps each sequence in the training dataset to a small set of latent factors that represent the parameters of a multi-dimensional Gaussian distribution. During training, we encode, sample at random from these distributions (one for each sequence), and finally pass the samples through the decoder. The training objective for the networks is to reproduce the original sequences from the decoded samples. VAEs are one of many types of models referred to as "generative," because once trained they can produce new and unique samples that share the properties of the training dataset.

We first assessed how well our model fit primary and secondary relationships in our training data. For any multiple sequence alignment, the variability at a given location can be represented as a categorical distribution over amino acids. Alignment averages of the training data and random samples from the model matched exceptionally well (Fig. 1C, Pearson's ${R}^{2}$ of 0.99), suggesting that the VAE effectively captures site-wise conservation statistics in our training data. To assess pairwise conservation, we calculated the mutual information (MI) between all alignment locations for the training data (Fig. 1D, left) and random samples from the model (Fig. 1D, middle). In this context, MI measures how much is known about the amino acid at a particular alignment location if the amino acid at the second location is known. A linear fit to all pairwise MI values shows strong correlations (Fig. 1D, right, Pearson's ${R}^{2}$ of 0.99), indicating that the VAEs effectively capture pairwise correlations between positions across the entire alignment.

Based on these results, we expected that many of these sampled OTC sequences would be functional enzymes. However, the strong bias for microbial genomes in sequence databases results in a type of class imbalance, where the number of eukaryotic (and specifically mammalian) OTCs are in the minority. Since VAEs are trained to reproduce their inputs, random sampling from an unweighted model is likely to result in prokaryotic-like sequences that might be less suitable for therapeutic applications due to the potential immunogenicity of highly mutated sequences relative to human wildtype.

We addressed the issue of class imbalance in two ways. First, we trained both an unweighted VAE and a VAE where the training data was sampled to favor human-like sequences (the weights are inversely proportional to the Hamming distance between each sequence and hOTC). Previous work has suggested that the distance between latent space encodings (${Z}_{dist}$) is correlated with sequence distance^18,24,40^. To address the question of whether a weighted model might better represent the sequence space most closely related to the human enzyme, we calculated the correlation between these measures in both of the models (Fig. 1E). The unweighted VAE exhibited similar correlation than the weighted VAE when comparing all training sequences (Pearson's ${R}^{2}$ of 0.29 vs 0.25, Fig. 1E, solid lines), but lower correlation when the comparison was restricted to the closest 5% of sequences (Pearson's ${R}^{2}$ of 0.56 vs 0.81, Fig. 1E, dashed lines). The lower overall correlation for all sequences reflects the much larger diversity of prokaryotic sequences in general. This result suggests that the weighted model better captures relationships in the sequence space surrounding hOTC, and we used it for all sequences generated in this work.

**Figure 2:**
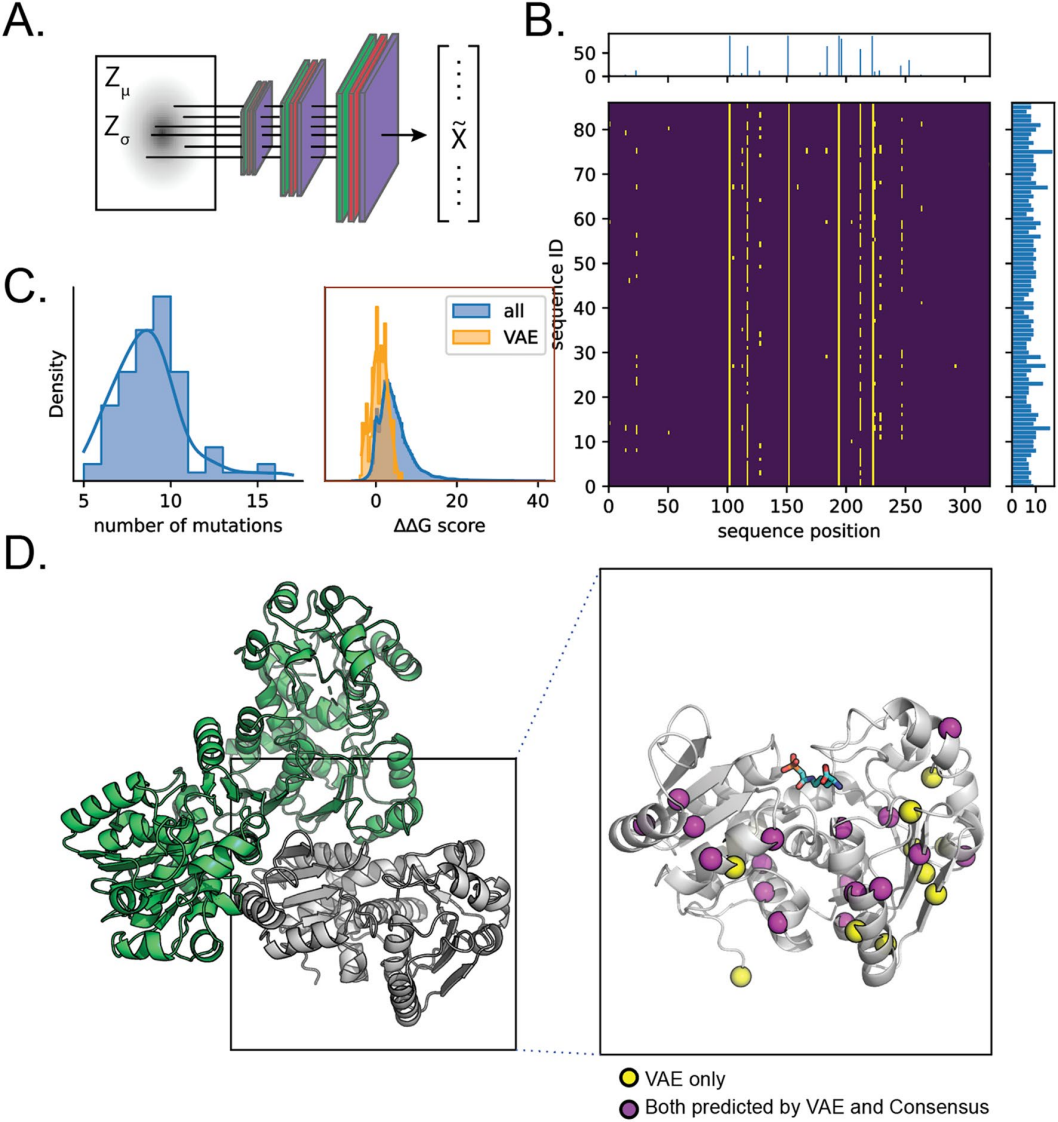
Sampling near-human OTCs. (**A**) Sampling schematic. By fixing $\upmu$ to be ${\mathrm{Z}}_{\upmu }$ OTC and progressively increasing $\upsigma$, we obtained 87 mutants with minimal mutations with respect to hOTC. (**B**) Alignment of VAE OTCs highlighting mutation locations. Center, alignment of sequences ordered by mutation count. Yellow marks a difference with respect to hOTC. Right marginal, summed mutation counts for each sequence; top marginal, summed mutation counts, per position. (**C**) Summary metrics of mutations. Left, distribution of mutation count with respect to hOTC; right, distributions of predicted $\mathrm{\Delta \Delta }$ G values for all possible single substitutions and for the VAE substitutions only. (**D**) Mapping of mutation locations onto hOTC structure (PDB: 1OTH). A cartoon representation of a single hOTC monomer of the homotrimer is shown in the inset with the ornithine analog N-phosphonacetyl-L-ornithine shown in cyan sticks. Mutation locations predicted by the VAE model only are shown in yellow spheres, and those predicted by both VAE and consensus models are in purple spheres.

Secondly, instead of randomly sampling from the latent space of our model or interpolating between sequences, we decided to encode hOTC and sample repeatedly from its encoded distribution (Fig. 2A). By scaling the variance of this distribution, we could exploit the close relationship between ${Z}_{dist}$ and sequence distance and limit the number of mutations relative to hOTC. This method yielded 87 unique near-hOTC variants (with an average of > 98% and no less than 95% identity) with mutations spanning the length of the gene at 32 distinct locations (Fig. 2B). The variants on average contained 8 amino acid substitutions relative to hOTC (Fig. 2C); these changes were distributed more or less evenly between the core and surface of the protein (Fig. 2D). Interestingly, none of the substitutions suggested by the VAE model are in the OTC active site (i.e., are known catalytic residues or within 5 Å of the substrate).

As an engineering control, we built a variant library based on consensus sequence design. Using the consensus sequence from our 3818-member OTC sequence dataset, we identified 18 amino acid differences between the hOTC wildtype sequence and the consensus. We encoded these amino acid substitutions into a fully combinatorial library of ~ 10^7^ variants and randomly selected 91 unique variants after transformation in *E. coli*, colony picking, sequence verification, and filtering for quality and fidelity to the library design (see “Methods”).

### Bacterial growth assays with VAE and consensus variants

**Figure 3:**
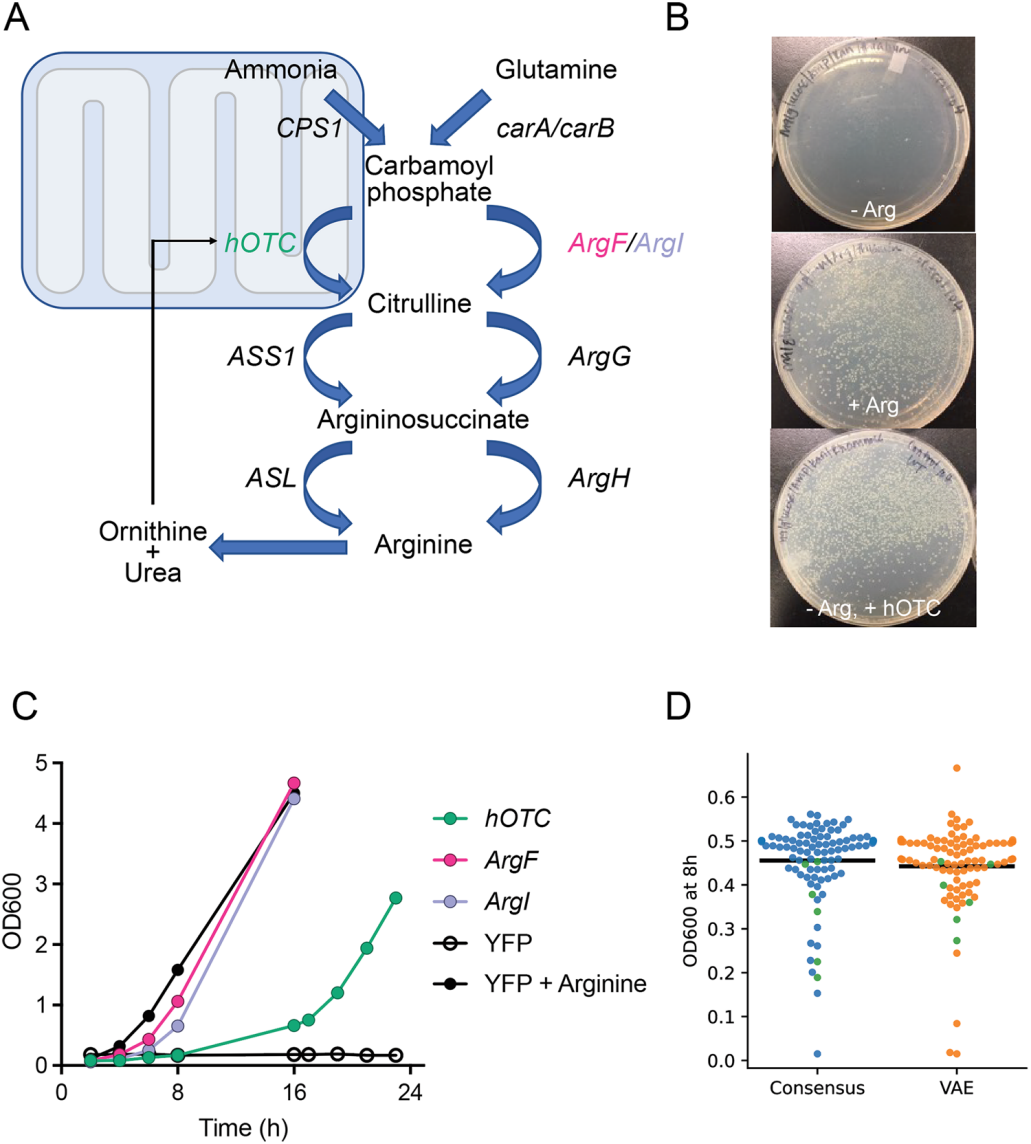
Auxotrophic growth assay for evaluation of hOTC variants. (**A**) Schematic of the urea cycle pathway in humans (left) and arginine biosynthesis in K12 *E.coli* (right). The first two enzymatic steps, catalyzed by CPS1 and OTC, in humans occur in the mitochondria. The remaining steps occur in the cytosol. Unlike humans, *E. coli* possess two genes each in place of CPS1 (*carA/carB*) and OTC (*argF/argI*). (**B**) Phenotype of the ∆*argF*∆*argI* double knockout *E. coli* strain. ∆*argF*∆*argI* cannot grow in the absence of arginine (top panel). Growth can be rescued either by arginine supplementation (middle panel) or by transformation of an external plasmid encoding hOTC (bottom panel). Black markings on the plates indicate growth media and plating conditions, see Methods for details. (**C**) Growth curves of the ∆*argF*∆*argI* strain expressing different OTC genes. The auxotroph was transformed with plasmids encoding YFP (negative control, black open circles), *hOTC* (green), *E. coli ArgF* (pink) or *E. coli ArgI* (purple) and growth was assessed in liquid medium lacking arginine. Solid black circles represent the growth rate of the strain when arginine is supplemented in the medium. (**D**) Growth rescue of the auxotroph by consensus and VAE hOTC variants. OD600 values at 8 h are shown for the auxotroph transformed with hOTC (green) or the variants (blue and orange for consensus and VAE, respectively). The hOTC data were collected multiple times within the same experiment and on separate days to show the spread in values. Only single data points were collected for the consensus and VAE variants; however, as seen by the data most of the variants have OD600 values greater than the highest measurement recorded for hOTC.

To distinguish between the VAE and consensus libraries in terms of function in biological context, we first screened all OTC variants for their ability to rescue growth of an *E. coli* arginine auxotroph. This strategy is feasible owing to the remarkable similarity between the urea cycle pathway in humans and the arginine biosynthesis pathway in *E. coli* (Fig. 3A, left and right respectively)^35,36^; four out of six urea cycle enzymes are conserved in *E. coli*, and deletion of any one (in case of *argG* and *argH*) or two (in case of *carA/carB* or *argF/argI*) of these enzymes produces a strain that cannot grow in the absence of arginine. Figure 3B (top panel) shows the phenotype of the ∆*argF*∆*argI* (i.e., ∆OTC) double knockout strain without arginine. Growth can be rescued upon arginine supplementation (Fig. 3B, middle panel) or by transforming the knockout strain with an external plasmid that supplies the missing gene, in this case hOTC (Fig. 3B, bottom panel).

To determine whether growth selections in the knockout strain can distinguish between enzymes of varying catalytic activities, ∆*argF*∆*argI E. coli* were transformed with recombinant plasmids encoding for different genes, and liquid cultures of the resulting strains were grown in the absence or presence of arginine. As seen in Fig. 3C, a negative control plasmid (YFP) lacking OTC activity cannot rescue growth of the auxotroph (black open circles) but does not interfere with rescue via addition of arginine (black solid circles). Human OTC is capable of rescuing growth at a doubling time of 3–4 h (green), while *E. coli* OTC-transformed bacteria (ArgF and ArgI in pink and light purple, respectively) show doubling times of 1.5–2 h, approaching the maximum growth rate of the strain as observed when arginine is added to the medium (black solid circles). These experiments demonstrate that *E. coli* growth selections recapitulate known differences in the catalytic efficiencies of human and *E. coli* OTC; the *E. coli* enzyme has been reported to have a five–tenfold higher *k*_cat_/K_M_ than hOTC^38,41–43^. Additional controls using known mutants of hOTC with lower catalytic activities^44,45^ confirm that growth rate can also be tuned down relative to hOTC (Fig. S1 in Supplemental Information). We note here that growth rate is only partially controlled by OTC catalytic activity; other parameters such as enzyme stability and translation-dependent expression may also be governing the observed doubling times. Nonetheless, these combined studies indicate that auxotroph’s growth rate can report directly on OTC enzymatic activity. We therefore predicted that OTC library variants of higher specific activity would promote faster doubling times than hOTC, enabling not only the identification of any individual variants with improved properties, but also observation of any library-wide differences between the VAE and consensus libraries, such as differences in the number of improved variants.

To test the prediction that variants of higher specific activity would promote faster doubling times, plasmids carrying unique VAE or consensus variants were individually transformed into the *E. coli* auxotroph and their impact on growth rate in defined media was assayed using a Biolector Pro microfermenter as described in the Methods section, with optical density at 600 nm (OD600) serving as a measure of growth rate. Figure 3D shows the OD600 of each culture after 8 h of incubation. Control cultures performed similarly to our previous observations (Fig. 3C) where growth rate increased in the order YFP < hOTC < ArgF with a fivefold difference in OD600 between hOTC and ArgF (data not shown). Remarkably, almost all VAE (84/87, orange) and consensus (87/91, blue) variants outperformed hOTC (green), promoting OD600 increases of 1.5–2.5-fold and, in some cases, approaching ArgF levels of growth rescue. Since a majority (~ 96% for both libraries) of the variants exhibited improvement relative to hOTC, we decided to recombinantly produce and characterize them all to understand differences between the two protein engineering approaches.

### Biochemical characterization of recombinant hOTC variants

As mentioned above, the growth rate of the *E. coli* auxotroph is controlled by multiple parameters, including protein expression (from improved translation or stability) and enzymatic activity. Upon observing that the VAE and consensus libraries performed similarly in the auxotrophic growth assays, we aimed to address two main questions. First, we sought to determine the fraction of VAE variants with improved specific activity and/or thermostability relative to hOTC (that is, improved catalytic rate normalized to enzyme concentration). Second, we asked if the VAE method provided any improvements in the hit rate relative to the consensus approach.

**Figure 4:**
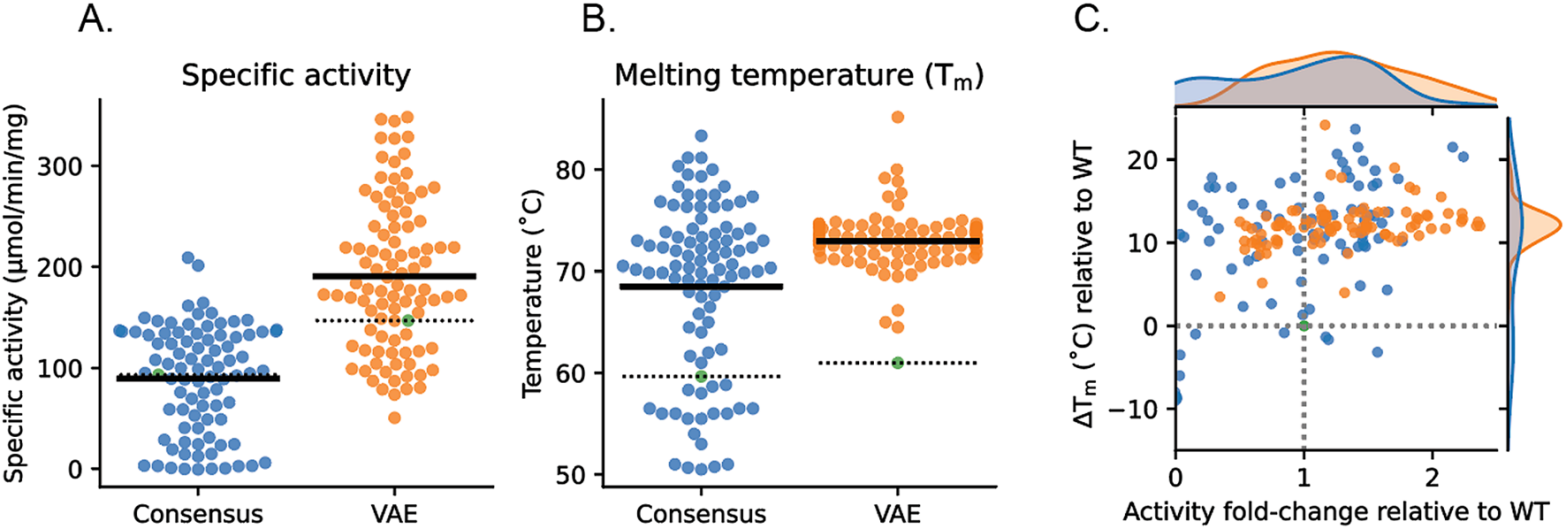
Specific activity and thermostability of recombinant protein for VAE and consensus hOTC variants. (**A**) Specific activity (μmol citrulline/min/mg protein) distribution of recombinant protein for VAE (orange) and consensus (blue) hOTC variants. Specific activity of hOTC is indicated by a green dot and the dashed line, and the mean specific activity by the solid line. Each point represents the average of three separate trials for a unique variant. (**B**) Melting temperature (T_m_ in °C) distribution of recombinant protein for VAE (orange) and consensus (blue) hOTC variants. T_m_ of hOTC is indicated by a green dot and dashed line, and the mean by the solid line. Each point represents the average of three separate trials for a unique variant. (**C**) Scatter plot of thermal stability (∆T_m_ in °C) vs specific activity (fold-change) for all OTC variants relative to hOTC. Histograms of each population are plotted on the top and left axes. Orange dots represent VAE variants, and blue dots represent consensus variants. Variants show a mix of improved specific activity, thermal stability, or both. The hOTC values are indicated by the dashed line and green dot.

To address these questions, all OTC variants (i.e., both VAE and consensus variants) were recombinantly produced, and activity and thermostability were characterized as described in the Methods section. The results of these experiments are summarized in Fig. 4. The specific activity of hOTC as reported in literature^38^ and measured herein is 100–150 μmol/min/mg. Figure 4A shows the distribution of OTC specific activity relative to hOTC (green circle, dotted lines). Fifty-eight out of the 87 VAE variants (~ 66%) showed > 20% improvements in specific activity, with the best variant catalyzing citrulline formation at ~ 350 μmol/min/mg (i.e., a 2.5-fold increase over hOTC). Seventeen of the variants (~ 20%) were within 20% of hOTC activity, and the remaining variants (~ 14%) had lower activity (by > 20%) than hOTC. By contrast, only 6 out of 91 (~ 6.5%) consensus variants improved activity > 20% relative to hOTC. Thirty-three consensus variants (36%) were within 20% of hOTC activity while 52 of the variants (57%) showed lower than 20% hOTC activity. Finally, comparison of the mean activity between the two libraries (solid black lines) indicates a statistically significant difference (*p* value < 0.0001); the consensus library on average has a mean activity that is 0.96-fold relative to hOTC while the VAE library shows a significant improvement with a mean activity of 1.4-fold relative to hOTC.

In contrast to the specific activity observations, all VAE variants exhibited improved thermostability with respect to hOTC (Fig. 4B, green circle, dotted line). The average melting temperature (T_m_) of the VAE library was 73 °C (solid black line), a 12 °C improvement over hOTC. Similarly, the consensus approach also yielded variants with improved thermostability; the average T_m_ of the consensus library was 68 °C (solid black line) or a 8 °C improvement over hOTC. However, unlike the VAE library, the consensus variants showed a wider spread of values between 60–80 °C.

To determine whether there was any relationship between thermostability and enzymatic activity in these sets of variants, T_m_ was plotted as a function of activity (fold change relative to hOTC; Fig. 4C). The dotted crosshair indicates the position of hOTC, and the VAE and consensus variants are shown in orange and blue, respectively. As described above and summarized in the Figure, both VAE and consensus approaches can be used to identify OTC variants of improved thermostability. However, the most striking difference between the two methods is seen when comparing the two quadrants in the upper half of the panel. The consensus approach yields more variants in the upper left quadrant (improved thermostability, but decreased specific activity), while the VAE method boosts the hit rate in the upper right quadrant (improved thermostability and improved specific activity). Thus, while both approaches produce OTC variants that improve activity to a similar level (> twofold over hOTC), VAE produces more of those variants (Fig. 4A,C).

Based on the results of these experiments, the 12 VAE sequences with highest specific activity were synthesized as codon-optimized mRNAs for in vitro evaluation.

### Performance of variant mRNAs in HepG2 mammalian cells

A key concern for development of therapeutic enzymes is the translatability of observations from bacterial growth models and biochemical characterization to in vitro mammalian cell models, in vivo animal disease models, and ultimately, patients. A complex array of biological factors can lead to poor translatability from model to model; for mRNA therapies, these factors include modified nucleotide chemistry, manufacturing process impurities, mRNA translation and decay, post-translational modifications of protein, cell-specific protein degradation pathways, and protein activity and stability. In the case of OTC specifically, the enzyme also requires co-translational translocation into the mitochondria and cleavage of the leader peptide to generate the active trimeric version^46^; similar issues of intracellular processing and localization are expected to be relevant for many other metabolic enzyme targets.

To determine whether recombinant OTC proteins with improved specific activity also show benefits when translated from mRNA in the cellular milieu, we synthesized codon-optimized mRNAs of the top 12 VAE variants. We do not anticipate the computational method (VAE vs consensus) to have an impact on translatability, since it is expected to depend on cell model factors that are independent of sequence engineering methods. We therefore chose to perform these experiments only with selected VAE hits.

**Figure 5:**
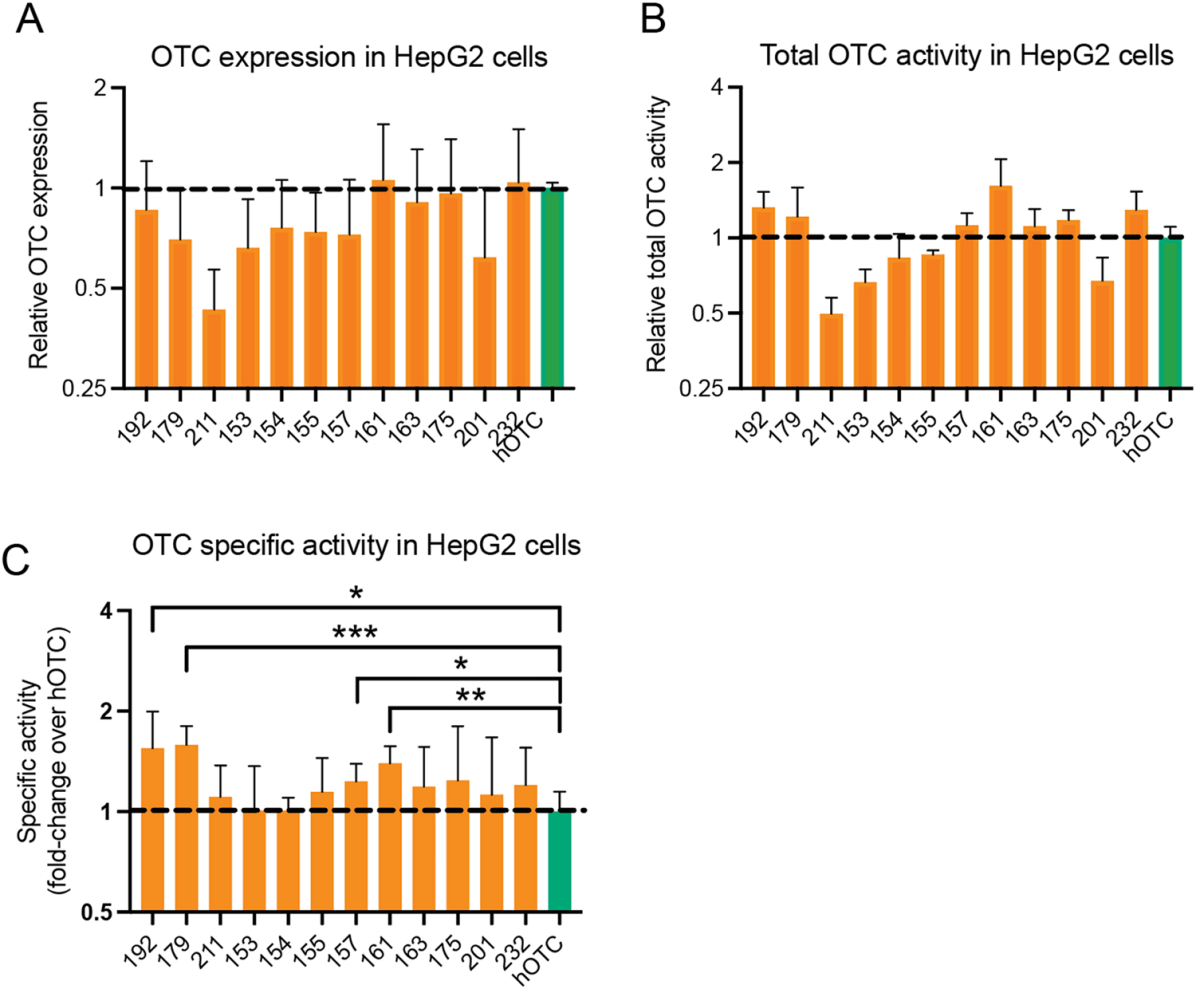
Performance of VAE hOTC variants in mammalian cells. (**A**) OTC expression in HepG2 cells transfected with OTC mRNAs. Expression was normalized relative to hOTC (black dashed line). Data points indicate the average values of three biological replicates and error bars indicate standard deviation. (**B**) Total OTC activity (nmol/min/mg total protein normalized to hOTC) in HepG2 cells transfected with OTC mRNAs. Data points indicate the average values of three biological and six technical replicates. Black error bars represent standard deviations. (**C**) OTC activity in HepG2 cells transfected with OTC mRNAs. Total activity (panel **B**, nmol citrulline/min/mg total protein) was normalized to the expression observed in panel A to determine specific activity of VAE OTCs. Asterisks represent significance of < 0.004 under Bonferroni-corrected t test.

The 12 VAE OTC mRNAs were synthesized as described in Materials and Methods and transfected into HepG2 cells, a liver cancer cell line that is typically OTC low/deficient^47^. OTC protein expression and activity were measured in total lysates one day after transfection. The data (in log2) are shown in Fig. 5A–C and represent the averages of three biological replicates (i.e. three separate transfections) and two technical replicates. Expression levels of the variants were on par or lower (0.5–0.75-fold) than that observed with hOTC (Fig. 5A, dotted black line). Despite these lower expression levels, seven of the variants showed the same or slightly improved total activity in cell lysates relative to hOTC (Fig. 5B), indicating higher specific activities. The total activity from panel B was normalized to protein expression from panel A, with the normalized values (specific activities) shown in Fig. 5C. As seen in the Figure, the specific activities of all OTC variants were 1–1.5-fold greater than that of hOTC (black dotted line) with four variants showing a statistically significant improvement in specific activity. Since OTC is only enzymatically active upon transport and proteolytic processing in the mitochondria^46^, this improved activity (and the absence of detectable pre-proteolyzed enzyme as judged by western blot, Figure S2) indicate successful translocation and processing of the OTC variants. These data thus confirm that the biochemical observations with the recombinant OTC proteins translate into mammalian cell-based systems where protein is made from mRNA.

### Identification of important mutation-function relationships

The VAE and consensus library variants have a range of specific activities, yet none of the represented substitutions are found in the active site (Fig. 2D). Furthermore, many variants show simultaneously improved activity and thermostability, defying the stability-activity trade-off assumption^48^. Attempts to understand the effects of individual mutations are confounded by the diversity of the sequence variants sampled from the VAE and consensus libraries, each of which contain between 5 and 25 mutations relative to the parental hOTC sequence (Fig. 2C).

We used regularized least squares linear regression to estimate the effects of individual mutations on specific activity and thermostability. This technique is a commonly used statistical tool for estimating contributions by the independent variables (e.g., individual mutations) to a set of observations of the dependent variables (e.g., specific activity and T_m_). Regularization is especially helpful when the data suffer from multicollinearity, whereby the independent variables are not fully independent.

**Figure 6:**
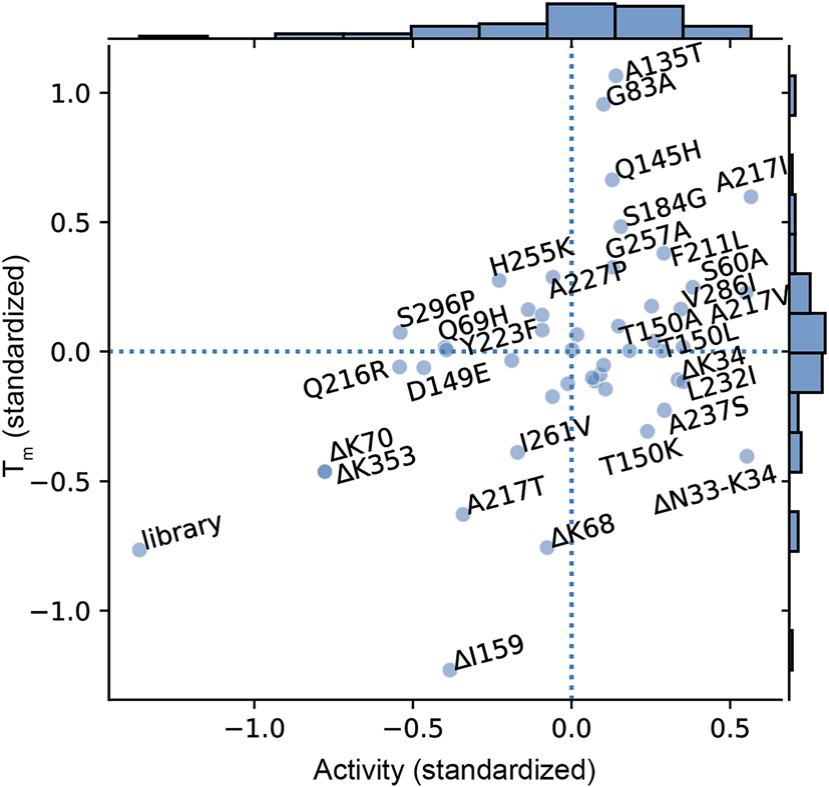
Effect of individual amino acid mutations on activity and stability. OTC activity vs. stability coefficients. The coefficients of linear ridge regression models relating amino acid mutations to activity and stability are plotted with standardized melting temperature (T_m_) and activity on different axes. Points in the upper right quadrant represent mutations that contribute to both stability and activity, whereas points in the lower left decrease both. Histograms of the coefficients are plotted on the right and top axes. Dashed lines represent the zero axes. Deletions are indicated by a delta (∆) prefix. The linear model is regularized by ridge regression.

The resulting coefficients from regularized Ridge regression^49^ of the single mutations are plotted with respect to specific activity and melting temperature (T_m_) in Fig. 6. A large positive coefficient for a mutation suggests that the mutation has a large positive effect on the measurement, whereas a negative coefficient suggests an anti-correlated effect. Deletions at K70, I159, and K353 have deleterious effects on enzymatic activity, but the N33-K34 double deletion appears to improve activity. With the exception of a few particularly harmful mutations, most substitutions appear to be neutral or beneficial to activity. G83A, A135T, Q145H, and A217I correlate positively with stability. Nearly all of the deletions appear to decrease stability.

The “library” coefficient captures experimental trial-specific differences in the measurement, as the consensus and VAE libraries were assessed in separate trials. For both T_m_ and activity, the negative coefficient for “library” suggests that the experimental bias partially explains the result, wherein the consensus library experiments yielded lower values than the VAE experiments. Figure 4A tells a similar story, in that the specific activity of the hOTC control is lower in the consensus experiment than in the VAE experiment. However, when normalized to hOTC, the VAE library has higher average activity relative to the consensus library.

Several single mutations either mutually increase specific activity and melting temperature (e.g., A217I, A217V, S60A) or decrease both properties (e.g., A217T, and K70 and K353 deletions). A few mutations seem to affect one property while being neutral to the other (e.g., G83A, A135T, Q145H, S184G, G257A for T_m_, and K34N, D149E, T150L, Q216R for activity). Note that A135T, Q145H, S184G, and G257A are also highly conserved consensus substitutions among the OTC homologs (Fig. S6). Finally, a few substitutions seem to trade off one property for the other (e.g., T150K, H255K, N33-K34 double mutant). Taken as a whole, however, specific activity and melting temperature do not appear to be globally anti-correlated, as might have been expected if the sequences were close to optimal with respect to both high specific activity and high melting temperature.

**Figure 7:**
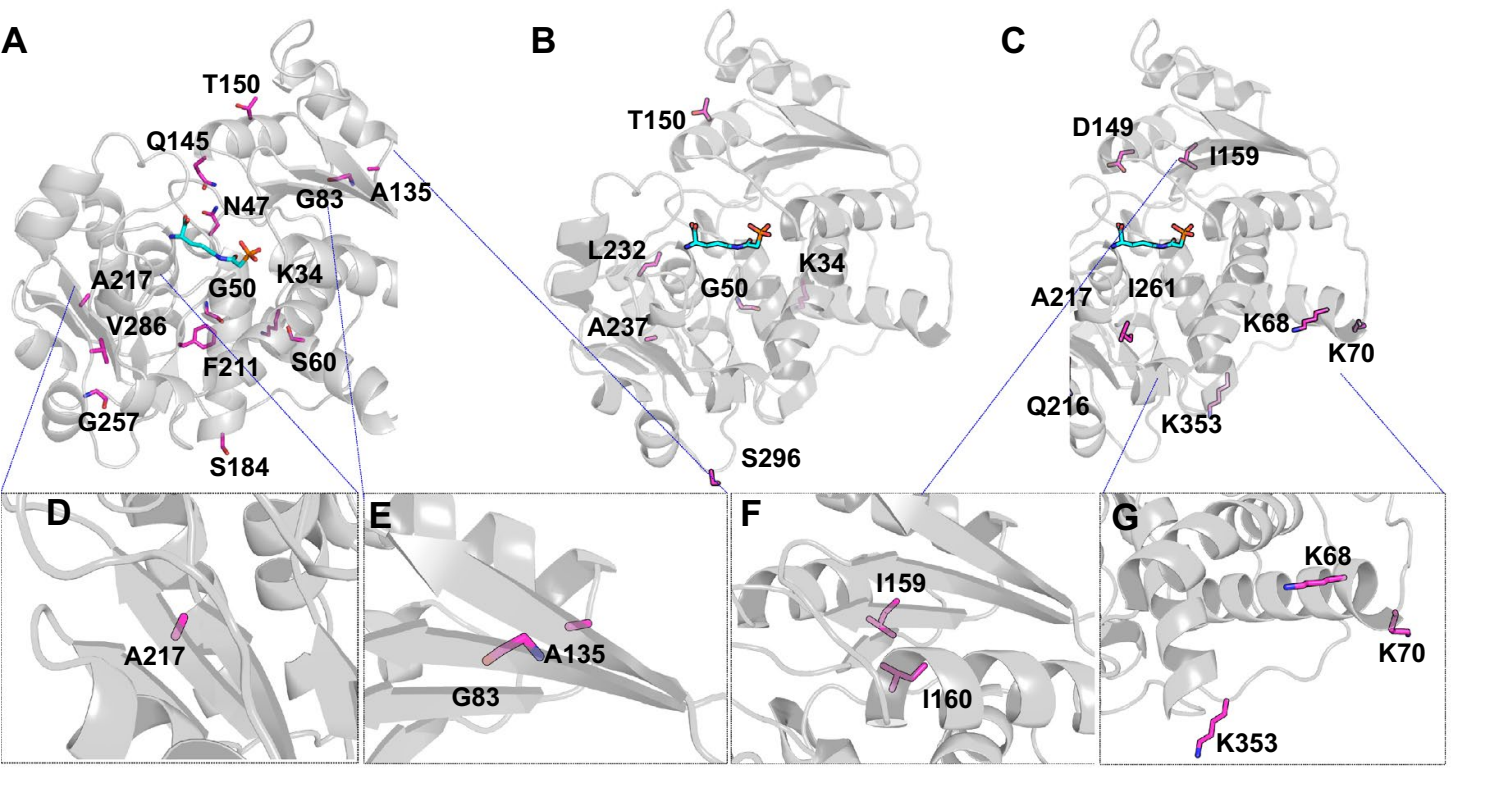
Structural mapping of important individual residues onto hOTC monomeric structure. A structure of the hOTC monomer (white cartoon) bound to a substrate analog (N-phosphonoacetyl-L-ornithine, cyan sticks) (PDB: 1OTH) is used to highlight amino acid positions mutated in the VAE-sampled sequences (magenta sticks). (**A**) Amino acid positions of mutations that have a beneficial/positive effect on T_m_ and specific activity. (**B**) Amino acid positions of mutations that have a beneficial/positive effect on either T_m_ or specific activity, but not both. (**C**) Amino acid positions of mutations that negatively affect T_m_ and specific activity. (**D**–**G**) Close-up views of A217, A135 and G83, the small beta-sheet consisting of I159 and I160 and the three important lysines at K68, K70 and K353.

Several mutations of interest are highlighted on the OTC crystal structure in Fig. 7. OTC seems particularly sensitive to mutations at A217. A217 is in a beta sheet that is buried in the hydrophobic core of the protein (Fig. 7), so mutations to hydrophobic Val or Ile may be more space filling than Ala and more compatible with the OTC structure than polar Thr. A135T and G83A have the most positive effects on melting temperature. A135 is found at the C-terminal cap of a helix at the trimer interface, and G83 is on a nearby beta-sheet (G83 C-alpha is roughly 4 Å from the A135 C-beta). Computational ∆∆G predictions suggest that A135T and G83A are energetically favorable (negative ∆∆G, Fig. S8), and Gly is considered to be a poor beta sheet-forming residue^50^.

Though most of the deletions assessed here are deleterious, the K34 single deletion and N33-K34 double deletion appear to enhance activity (the N33 deletion has a neutral effect on activity and T_m_). K34N also appears to enhance activity. N33 and K34 are the N-terminal residues of the active enzyme after proteolytic processing to remove the mitochondrial leader peptide. K68, K70 and K353, three harmful deletions, are surface-exposed, the first two at the end of a helix and the latter at the C-terminus. I159 is in a short, buried beta strand, so removing it further shortens the beta strand, though the Ile sidechain is effectively replaced by I160.

## Discussion

In this work, we trained a machine learning model on a dataset of natural OTC sequences, from which we then sampled tens of near-human variants. We also used the same set of OTC sequences and a consensus-based approach to generate a control library of similar size. Sequences from both libraries supported improved growth in a growth selection with auxotrophic bacteria; the large percentage (~ 96%) of VAE sequences demonstrated to have improved properties in the auxotrophic bacteria suggests that this screening step might be omitted in future metabolic enzyme engineering efforts. Because the key difference between the VAE and consensus libraries is that the VAE library incorporates residue-residue correlations, a comparison of the functional properties of these two libraries provides an approach to understanding the role of such correlations in OTC stability and catalytic function.

The VAE library, but not the consensus library, had a mean specific activity higher than the human wild-type OTC; this difference is modest (1.4-fold), but statistically significant (p value < 0.0001; Fig. 4). However, the variance in catalytic activity was comparable between the two libraries (0.32 for the consensus library and 0.3 for the VAE library; Fig. 4). The enhanced mean activity observed for the VAE library suggests that the epistatic interactions captured in this library contribute to increased catalytic rate. However, the observation that the VAE and consensus libraries exhibit comparable variance in activity suggests that the residues involved in epistatic interactions do not dominate in their contributions to catalytic activity. If those interactions made the bulk of the contributions to catalytic activity, the consensus library would have been expected to sample a smaller range of values for catalytic activity than the VAE library, since it would be missing the largest positive or negative contributions.

With respect to thermal stability, both consensus and VAE approaches yielded libraries with substantially elevated average melting temperatures: a 8 C° improvement for the consensus library and a 12 C° improvement for the VAE library. However, although the mean T_m_s were similar, the consensus library contains more variants at the extremes; the variance in T_m_ is 35.5 for the consensus library and 4.4 for the VAE library. Consensus mutation strategies have previously been shown to be effective in generating protein variants with comparably high levels of stabilization^7,14,51^. The similarity in thermal stability between the libraries may reflect the fact that the VAE library is highly enriched in consensus mutations and suggests that the effects on stability are mostly accounted for at the individual residue level, rather than epistasis between residues.

In contrast, the difference in the spread of thermal stability in the OTC libraries is consistent with two countervailing selective pressures operating at the level of residue-residue correlations. First, there may be a general selective pressure to enrich for individual mutations which stabilize the protein. These may enhance the overall evolvability of the sequence, as they would provide a permissive context for subsequent mutations that improve other functions at the cost of stability. This would manifest itself in the consensus mutations exhibiting increased thermal stability, as observed. However, there also may be a countervailing selective pressure against unchecked upward drift of thermal stability; extremely stable enzymes may have reduced catalytic rates and/or a prohibitively high energetic cost of degradation. The push and pull of these forces over time would select against variants at the extremes of the stability spectrum and such selection would be encoded in residue-residue correlations. Accordingly, we see a tighter distribution of thermal stabilities in the VAE library, which preserves these correlations, than in the consensus library, which does not.

Taken together, these results support the conclusion that different residues contribute differentially to changes in stability and catalysis, as expected. Perhaps surprisingly, however, no trade-off between stability and catalysis is observed; this might be due to mutational effects on these properties generally being uncorrelated in this system, and/or to a selection against mutations with correlated effects in the starting set of natural sequences. The results also suggest that interactions between residues contribute differentially to thermal stability and catalysis. However, we were limited in the number of variants we tested, and further experiments and analysis will be needed to fully investigate the physical/mechanistic basis of library mutations on OTC stability and catalytic activity.

The results of this study suggest future applications of VAE models both in enzyme engineering and in the study of enzyme structure–function relationships. For enzyme engineering, the use of the VAE model enables design of a library that yields improved variants (up to a ~ twofold increase in catalytic activity with greater thermal stability) with a library size of only ~ 10^2^, indicating that the VAE model is sampling a highly functional region of sequence space. This characteristic of the VAE sequences also may enable detailed structure–function studies of epistatic contributions to enzyme catalysis in OTC and other enzymes. Because the VAE generates a potentially large number of variants with residue-residue correlations, highly correlated mutations can be characterized to determine functional epistasis, potentially allowing the mapping of functional interaction networks in the enzyme structure.

By capturing higher-order co-evolutionary relationships, generative machine learning models have the potential to aid in understanding protein structure and function in novel ways. Training on natural sequences implicitly limits exploration of sequence space, greatly improving the odds of finding variants. A posteriori examination of the relationship between sampled mutations and protein function can generate specific testable hypotheses for experimental biologists. Combined with large-scale unsupervised approaches^18,20^ and multiple rounds of sampling, generative models are poised to usher in an exciting new generation of protein engineering approaches.

## Methods

### Materials

The ∆*argF*∆*argI* double knockout *E. coli* strain was constructed by the Coli Genetic Stock Center at Yale. ATUM Bio supplied the backbone vector (pD884) for cloning. Terrific Broth (TB), M9 minimal medium, carbenicillin (100 mg/mL), kanamycin (50 mg/mL), and Luria broth (LB)/agar plates containing carbenicillin/kanamycin were obtained from Teknova. Ampicillin, L-rhamnose monohydrate, triethanolamine hydrochloride, sulfuric acid, acetic acid, lithium carbamoyl phosphate dibasic hydrate, L-ornithine monohydrochloride, diacetyl monoxime and L-citrulline were purchased from Sigma Aldrich. Thermo Fisher Scientific provided B-PER Complete Bacterial Protein Extraction Reagent, HisPur Ni–NTA spin plates, antipyrine, SYPRO Orange (5000X) dye, Lipofectamine 2000 and the bicinchoninic acid (BCA) assay kit. Novus Biologicals provided the rabbit polyclonal OTC antibody (NBP1-87408) for western blot analysis. All oligonucleotides in this work were purchased from IDT and Q5 DNA polymerase (NEB) was used for all PCR reactions.

### Training dataset generation

The initial set OTC homologs was obtained by a BLASTp search of the “nr” database (“non-redundant protein sequences”) with canonical human OTC (UniProt ID: P00480) as the query sequence. Default parameters were used (BLOSUM62 similarity matrix, expect threshold 10, gap open penalty 11, gap extension penalty 1, no filter, 5000 result limit). The resulting sequences were then re-aligned to the parental hOTC sequence using the multiple sequence alignment (MSA) software tool MAFFT^52^ (v7.407).

For OTC and other urea cycle enzymes, the evolutionary history of homologous enzymes that began with de novo arginine biosynthesis in bacteria and archaea provides an extensive set of sequences for use in modeling. This sequence family is highly diverse; we attempted to filter out structurally dissimilar homologs by assuming that sequences that introduce large gaps in an alignment with hOTC have dissimilar structures. We score the “gappiness” of each sequence *i* in our alignment as

$${s}_{i}={\sum }_{j\in {G}_{i}}{f}_{i}^{j}$$

$${f}_{i}^{j}=\left\{\begin{array}{cc}{N}_{\text{gap}}^{j}/N,& if\, {N}_{gap}^{j}/N>w\\ 0,& {\text{otherwise}}\end{array}\right.$$

where *j* is the alignment column index, ${G}_{i}$ is the subset of column indices at which sequence *i* has a gap letter, *N* is the number of sequences in the alignment, ${N}_{\text{gap}}^{j}$ is the number of gap letters in column *j*, and $w\in \left[\mathrm{0,1}\right]$ is the gap count weight for thresholding if a column should be scored as a gap or not.

Each sequence was assigned a gappiness score with $w=0.9$, and those sequences *i* with ${s}_{i}>10$ (1011 sequences) were removed from the alignment. An additional 162 sequences were removed because their ungapped sequence lengths were too short (< 290 amino acids) or because they lacked “Ornithine” in their Genbank sequence description. Finally, the sequence alignment was sliced to remove any remaining gaps in the aligned hOTC sequence.

### Neural network modeling and variant sampling

Variational autoencoders are a recently developed class of probabilistic latent variable models that have been applied to a wide variety of data modalities, including biological sequences. These models assume that the data are the result of a random process involving continuous unobserved variables $z\in Z$. Generation of data involves first sampling a particular ${z}_{i}$ from a prior probability distribution ($p\left(z\right)$), and then a data point ${x}_{i}\in X$ is generated from a conditional distribution ${p}_{\theta }\left(x|z\right)$, where $\theta$ represents the parameters of the decoding neural network. In general, we would like to compute

$$\mathrm{log}{p}_{\theta }\left(x\right)={\text{log}}\int {p}_{\theta }\left(x|z\right)p\left(z\right)dz$$

but for most $z$, ${p}_{\theta }\left(x|z\right)$ will be nearly zero and comprehensively sampling from p(z) is intractable. Instead, VAEs approximate $\mathrm{log}{p}_{\theta }\left(x\right)$ with the Evidence Lower BOund (ELBO):

$$\mathrm{log}{p}_{\theta }\left(x\right)\ge {\mathbb{E}}_{q}[\mathrm{log}{p}_{\theta }(x|z)]- {D}_{KL}\left({q}_{\phi }\left(z|x\right)\parallel p\left(x\right)\right)$$

Here, ${q}_{\phi }$ is a family of normal distributions approximating $p\left(z|x\right)$ represented by encoding neural network, and ${D}_{KL}$ is the Kullback–Leibler divergence. The variational autoencoder simultaneously learns ${p}_{\theta }$ and ${q}_{\phi }$ by minimizing the ELBO via gradient descent^39^.

OTC sequences were represented as two dimensional one-hot matrices, where the size of the first dimension was 322 (the length of the OTC alignment), and the second was 21 (20 amino acids + gap). Weighted sampling with replacement was used to preferentially select training data for each batch based on the similarity to hOTC. These weights were calculated by raising the fraction of sequence identity to the 2.25 power, which has the effect of preferentially down-weighting distant sequences. We used a 90/10 training/test split and a batch size of 128.

Our VAE consists of two convolutional neural networks (an encoder and a decoder). The encoder contained three consecutive blocks of two-dimensional convolution, batch normalization, ELU non-linearities, and max pooling. The number of filters in the encoder blocks were 128, 96, and 64 respectively, and the outputs were pooled two-fold in the length dimension only. Similarly, the decoder contained three consecutive blocks of two-dimensional convolution, batch normalization, ELU non-linearities, and nearest neighbor upsampling (in the length dimension only) with a final soft-max layer to normalize over the amino acid dimension. The number of filters in the decoder blocks were 64, 96, and 128 respectively. We used a latent space of dimension 64. Network weights were initialized with the default “Kaiming normal” approach^53^. All code was written in Python 3.6 and models were specified in the PyTorch framework (code available upon request).

The two terms of the right-hand side of the ELBO above correspond to the cross entropy and divergence components of the total VAE loss we minimize. Following a common procedure from the literature, we annealed the divergence loss with a scalar $\beta$ that varied from 0 to 1, increasing linearly on every epoch of training. We minimized the loss using the PyTorch Adam optimizer until test loss convergence, which took 30 epochs.

To sample near-human variants from the VAE for testing, we first encoded the hOTC sequence, resulting in a set of means and variances. We used these means and scaled the variances to sample Z values that we passed through the decoder and converted to biological sequences by picking the amino acid with the highest probability at each location (an argmax over the amino acid dimension). A variance of near 0 thus resulted in the maximum a posteriori Z value for hOTC, which when decoded resulted in a sequence with the minimal number of mutations relative to hOTC. We then repeatedly increased the variance and sampled until we reached an upper limit of mutations in any given variant of 16, or ~ 5%. After removing duplicates, this resulted in the 87 variants tested in this work.

### Design and sampling of consensus variant library (engineering control)

We compared our VAE-enabled data-driven protein design approach to consensus design, a simple and effective data-driven approach that derives the consensus sequence from an alignment of homologous sequences, and then suggests substitutions that convert the target sequence (hOTC) to the consensus^7^. The OTC consensus sequence was derived from the same multiple sequence alignment used to train the VAE, and the resulting consensus substitutions were converted into a combinatorial library using degenerate codons optimized by SwiftLib^54^. The consensus combinatorial library (~ 10^7^ possible variants) was assembled from degenerate codon oligonucleotides using overlap extension PCR and Gibson assembly as described in the Automated Gene Synthesis Platform section.

The library was transformed into the ∆*argF*∆*argI* double knockout *E. coli* strain, plated on LB agar plates containing 100 µg/ml carbenicillin and 50 µg/ml kanamycin, and incubated 16 h at 30 °C. 192 transformants were picked from the resulting colonies, inoculated into 2-ml 96-deep well plates containing 1 ml TB animal-free media (Teknova) containing 100 µg/ml carbenicillin, and incubated for 16 h at 37 °C with shaking at 300 rpm. Plasmid DNA was isolated from each clone via mini-prep and sequence confirmed using Sanger sequencing. Ninety-one sequence-verified consensus clones were selected for further testing.

### Information theoretic analysis of VAE model

To assess the ability of our VAE model to capture first-order sequence conservation and second-order sequence correlations in the evolutionary dataset, we use two metrics from information theory: entropy and mutual information. Entropy, also called information entropy or Shannon entropy, is a measure of information content in a random variable (in our case, a column in our sequence alignment) and is defined as

$${H}_{j}=-{\sum }_{a\in A}{\tilde{p }}_{j}^{a}\mathrm{log}{\tilde{p }}_{j}^{a}$$

$${\tilde{p }}_{j}^{a}={n}_{j}^{a}/N$$

where *j* is the alignment column index, ${n}_{j}^{a}$ is the number of observations in the *j*th column of letter *a* from the amino acid alphabet *A*, and *N* is the number of sequences in the alignment. The value ${\tilde{p }}_{j}^{a}$ is intended as a rough approximation of the probability distribution of A, though we make no statistical assumptions or rare event corrections.

Similarly, mutual information serves as a proxy for residue-residue correlations. For a pair of sequence alignment column indices *j* and *k*, the mutual information (MI) is defined as

$${I}_{j,k}={\sum }_{a\in A}{\sum }_{b\in A}{\tilde{p }}_{j,k}^{a,b}\mathrm{log}\left(\frac{{\tilde{p }}_{j,k}^{a,b}}{{\tilde{p }}_{j}^{a}{\tilde{p }}_{k}^{b}}\right)$$

$${\tilde{p }}_{j,k}^{a,b}={n}_{j,k}^{a,b}/N$$

where ${n}_{j,k}^{a,b}$ is the number of occurrences of a pair of letters *a* and *b* (paired in a single sequence) in columns *j* and *k*. Alternatively

$${I}_{j,k}={H}_{j}+{H}_{k}-{H}_{j,k}$$

where ${H}_{j,k}$ is the joint entropy

$${H}_{j,k}=-{\sum }_{a\in A}{\sum }_{b\in A}{\tilde{p }}_{j,k}^{a,b}\mathrm{log}{\tilde{p }}_{j,k}^{a,b}$$

### Automated gene construction platform for synthesis of hOTC variants

The target set of 87 amino acid sequences sampled from the VAE were reverse translated to DNA sequences that were codon optimized for *E. coli* K12 strains. These DNA sequences were then converted into a library of short overlapping oligonucleotide fragments that could be assembled into the target sequences by overlap extension PCR. Oligonucleotide overlaps were globally optimized over the 87-member sequence alignment, with consideration for GC content and hairpin and tandem repeats. In addition, the oligonucleotide library was designed to avoid diversity in the overlap regions, and the terminal oligonucleotides also include overlaps for Gibson assembly^55,56^ into the pD884 (ATUM Bio) *E. coli* plasmid.

To clone each OTC variant, we utilized an internal, automated gene synthesis method. Eighty-seven explicit combinations of oligos, which include complementary overhangs to allow for assembly into full length dsDNA fragments, were pooled using a Hamilton liquid handler. Each oligo pool was then assembled into full length dsDNA fragments and amplified using PCR. As the oligos contain errors in sequence, an enzymatic error correction step was included during synthesis of the explicit dsDNA fragments. After synthesis, each fragment was cloned into plasmid backbone using NEB’s 2X HiFi Gibson Assembly master mix. DNA products from the Gibson assembly reactions were individually transformed into NEB 10β for isolation of clones and sequence confirmation using Sanger sequencing.

### Growth assays in *E. coli* auxotroph

Purified plasmids carrying WT hOTC, VAE and consensus hOTC variants were transformed into the ∆*argF*∆*argI* double knockout *E. coli* strain. Single colonies were inoculated into 2-ml 96-deep well plates containing 1 ml LB media containing 100 µg/ml carbenicillin and 50 µg/ml kanamycin, and incubated for 16 h at 37 °C with shaking at 300 rpm. After cultures were grown to saturation, 1 µl of each culture/well was transferred into 2-ml 96-deep well plates containing 0.5 ml M9 minimal media with 20 g/l glucose, 40 µM arginine, 100 µg/ml carbenicillin and 50 µg/ml kanamycin, and incubated for 24 h at 37 °C with shaking at 300 rpm. Following growth of these seed cultures to saturation, they were used to inoculate Biolector Pro 48-well culture plates containing 0.8 ml M9 minimal media with 20 g/l glucose, 100 µg/ml carbenicillin and 50 µg/ml kanamycin to an initial OD600 of 0.05. Initial growth curves (Figure S3) showed that cultures hit logarithmic phase after 6–8 h of growth at 37 °C, 1500 rpm. Subsequently, single-time point measurements of each culture were recorded using a microplate spectrophotometer. Strains with plasmid-borne Human wild-type OTC (hOTC) and *E. coli* ArgF were analyzed simultaneously as controls.

### Recombinant expression and purification of OTC variants

Variant VAE and consensus OTCs were over-expressed as N-terminal hexahistidine (His_6_)-tagged proteins under the control of a rhamnose promoter. Protein expression steps were performed in 2-mL deep 96-well plates. Briefly, plasmids encoding the variants were transformed into BL21 chemically competent cells. Cells were diluted into fresh LB medium + ampicillin (100 μg/mL) and grown overnight at 37 °C with shaking. Starter cultures were used to inoculate (1:100 dilution) fresh TB medium containing ampicillin and grown at 37 °C until an OD600 of ~ 0.8 was reached, at which point L-rhamnose (0.05% w/v final) was added to induce protein expression. Growth was continued for an additional 4 h, and cells were harvested by centrifugation (3000×*g*, 10 min). Cell pellets were lysed in B-PER Complete and His_6_-tagged OTC proteins were purified using 96-well Ni–NTA agarose-containing spin plates following the manufacturer’s protocol. Final protein concentrations were determined using A280 and confirmed by SDS-PAGE analysis (Figure S4).

### Enzyme activity assays

Recombinant OTC variants were measured for their ability to catalyze citrulline formation from ornithine and carbamoyl phosphate using the method of Ceriotti^57^. Assays were performed in 96-well plates in a total volume of 100 μL; reaction mixtures contained 5 nM OTC enzyme, 1 mM carbamoyl phosphate and 1 mM ornithine in 270 mM triethanolamine HCl, pH 7.7. Samples were incubated at 37 °C for 5–15 min and quenched with 100 μL of a color reagent (two parts antipyrine/H_2_SO_4_ and one part 6% diacetyloxime in 5% acetic acid). Samples were incubated in the dark (16 h at room temperature), exposed under a fluorescent light equipped with a 22 W single-bulb lamp (20 min at 45 °C), and measured for absorbance at 490 nm using a BioTek Cytation 3 multi-mode reader. Citrulline dilutions of known concentrations were simultaneously treated with the color reagent and processed as described above. This standard curve was used to calculate specific activity of the OTC variants (μmol citrulline/min/mg protein).

### Differential scanning fluorimetry (DSF) assays

Protein stability as a function of temperature was measured via observation of the interaction of the hydrophobic SYPRO Orange dye with unfolded protein^58^. OTC variants (~ 0.25 mg/mL), SYPRO Orange (10X final concentration) and 100 mM triethanolamine HCl (pH 7.7) were combined in a volume of 25 μL in 384-well PCR plates. DSF assays were performed on a Bio-Rad CFX384 Touch Real-Time PCR Detection System. Temperature was increased from 25 °C to 90 °C in 0.5 °C increments. Samples were incubated for 5 s at each temperature prior to fluorescence measurement. T_m_ values were obtained from the top of the peaks in derivative plots (i.e., d(fluorescence)/dT vs temperature).

### Variant mRNA synthesis

OTC mRNAs were codon-optimized and synthesized as previously described^59^; mRNA synthesis utilizes in vitro transcription of linearized DNA templates by T7 RNA polymerase. The transcription reaction replaces all uridines with N1-methylpseudouridine and installs the 5ʹ and 3ʹ untranslated regions and the poly-A tail. OTC mRNAs were purified following 5ʹ cap addition.

### Activity of OTC variants in HepG2 cells

OTC mRNAs (0.25 μg) were transiently transfected into HepG2 cells (ATCC, HB-8065) seeded in 6-well plates using a 3:1 μL/μg ratio of lipofectamine 2000:mRNA. Cells were lysed after one day in 10 mM HEPES (pH 7.4) containing 0.5% Triton X-100, 2 mM dithiothreitol and 1X protease inhibitor cocktail. Total protein content in clarified lysates was determined using the bicinchoninic acid assay (Thermo Fisher). OTC protein expression was assessed by western blot analysis and activity was measured in 1–2 μg liver extracts using the colorimetric assay of Ceriotti^57^ as described above. Enzyme activity (expressed as nmol citrulline/min/mg total protein) was normalized to relative OTC expression as judged by densitometry analysis of the western blot. Statistical analysis of specific activity differences took multiple comparisons into account using Bonferroni’s correction.

### Statistical analysis of assay results to identify top individual substitutions

Linear regression analysis is a popular statistical tool for estimating contributions to a set of *observations* (i.e., sequence variants) of the dependent variables or *responses* (i.e., specific activity and Tm) by the independent variables or *predictors* or *features* (i.e., individual substitutions). A linear model is simply a weighted sum of $p$ predictors $\mathbf{x}={\{x}_{1}\dots {x}_{p}\}$

$$y={\sum }_{k}{\beta }_{k}{x}_{k}+\varepsilon$$

$$={x}^{\mathrm{\top }}{\varvec{\beta}}+\varepsilon$$

where ${\varvec{\upbeta}}={\{\beta }_{1}\dots {\beta }_{p}\}$ are the weights or *coefficients*, $y$ is the response, $\varepsilon$is the error, and $\mathrm{\top }$ indicates a vector transpose operation.

Ordinary least squares regression attempts to solve the following objective function given the linear model of coefficients $\widehat{{\varvec{\upbeta}}}=\{\dots {\widehat{\beta }}_{p}\}$ for $n$ observations of the response $\mathbf{y}={\{y}_{1}\dots {y}_{n}\}$ and $p$-dimensional predictors $\mathbf{X}\in {\mathbb{R}}^{n\times p}$:

$$\widehat{{\varvec{\beta}}}=\underset{{\varvec{\beta}}}{\mathrm{argmin}}{\Vert \mathbf{y}-\mathbf{X}{\varvec{\beta}}\Vert }_{2}^{2}$$

Our observations include all 87 sampled VAE sequences and 85 of 90 consensus library sequences after some initial filtering to remove entries with null data ($n=182$). For each of the two response variables for specific activity and melting temperature (T_m_), the triplicate measurements are averaged and standardized into z-scores:

$$z=\left(y-{\mu }_{y}\right)/{\sigma }_{y}$$

where ${\mu }_{y}$ and ${\sigma }_{y}$ and the mean and standard deviation of $y$, respectively.

The predictors are a set of binary categorical variables denoting presence (1) or absence (0) of a specific mutation (substitution or deletion). Because the VAE and consensus library variants were assayed separately, we also include an additional categorical predictor to indicate the assay group (i.e., VAE or consensus). The group predictor is 0 for any VAE observations and 1 for the consensus observations. The total number of predictors from the combined sampled VAE and consensus libraries is 48 (Fig. S6).

We apply multilinear regression with Ridge (L2-norm) regularization. Regularization is a useful technique that reduces overfitting by applying a constraint term to the minimization objective, e.g., a penalty on the magnitude of the weights. We apply Ridge linear regression to both the standardized specific activity and melting temperature measurements.

### Structural analysis of substitutions

Fixed-backbone changes in folding free energy due to mutation (∆∆Gs) were predicted using the Rosetta macromolecular modeling suite^60,61^. High-resolution crystal structures of hOTC with different substrates (PDB: 1OTH, 1EP9, 1FVO, 1C9Y) were prepared by removing water and ions and then minimizing with heavy-atom crystallographic constraints using the Rosetta constrained minimization protocol *minimize_with_cst*. These minimized models were assessed for quality by checking RMSD from the reference crystal structures and filtering those models without any steric clashes or other residue-level abnormalities highlighted by the Rosetta score function. Finally, all possible single substitutions in the prepared structures were scanned using the Rosetta *ddg_monomer* protocol. Results were tabulated and summarized using the Python Pandas package. Secondary structures were determined from the PDB secondary structure records and also by DSSP^62^. Relative solvent accessible surface area calculated using MDTraj^63^.

## Supplementary Information

Supplementary Information.
