# Supplementary material for: Therapeutic enzyme engineering using a generative neural network
Source: Sci Rep. 2022 Jan 27;12:1536. doi: 10.1038/s41598-022-05195-x (PMC8795449; doi:10.1038/s41598-022-05195-x)
Supplement: Supplementary file 1 — Supplementary Information. [file 41598_2022_5195_MOESM1_ESM.pdf]

Supplemental information

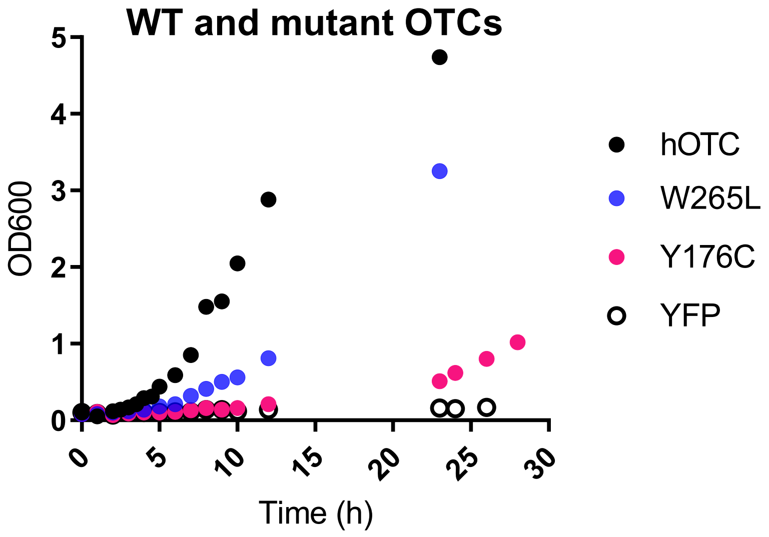

Figure S1. Growth rescue of the *E. coli* auxotroph by wild-type hOTC and variants of decreased catalytic activity. The auxotroph was transformed with plasmids encoding for YFP (open black circles, negative control), hOTC (black solid circles), W265L (blue solid circles, reported to have 50% of hOTC activity) and Y176C (solid pink circles, reported to have 20% hOTC activity).

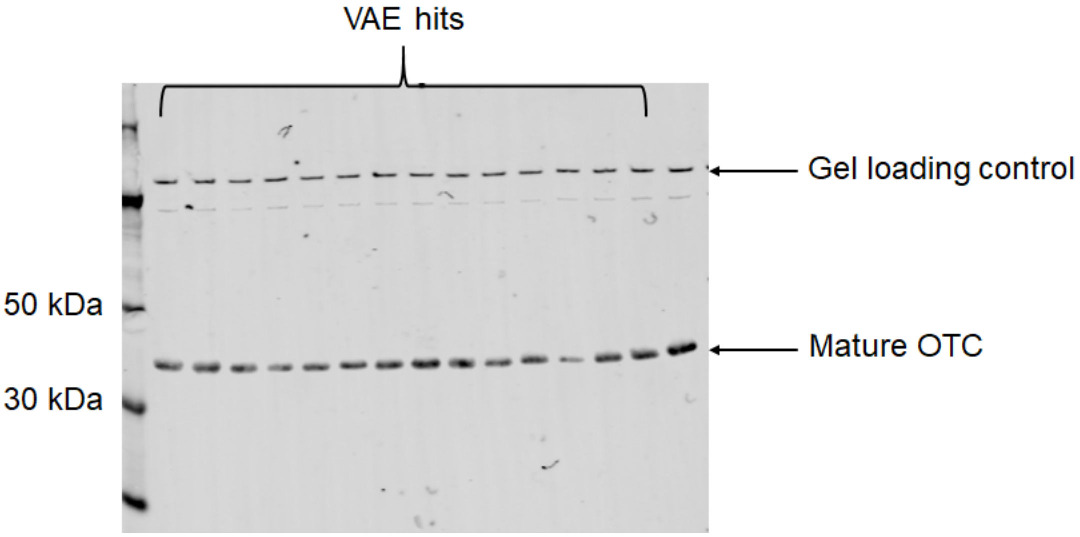

Figure S2. Western blot analysis of HepG2 cell lysates post transfection with OTC mRNAs. OTC migrates as a single band with no evidence of pre-proteolyzed enzyme. Blot represents the full-length image with no modifications.

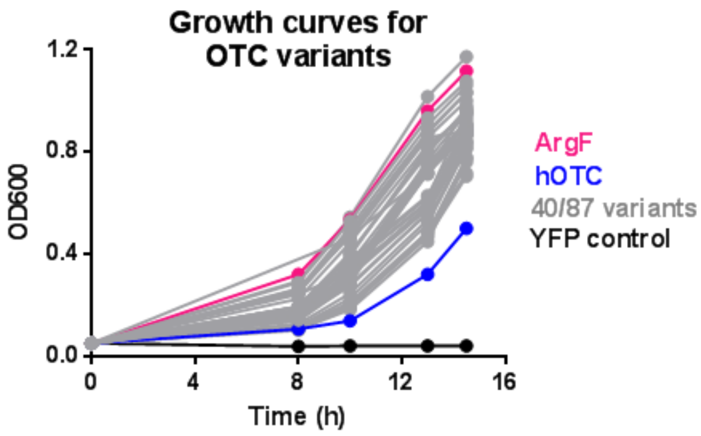

Figure S3. Liquid medium growth curves for 40 explicit hOTC variants. Plasmids encoding the protein variants were transformed in an *E. coli* auxotroph and tested for their ability to grow without arginine supplementation. All variants tested (grey) outperformed hOTC (blue) with some approaching *E. coli* ArgF (pink). Based on these observations, all subsequent growth curves with the hOTC and control libraries were analyzed at a single time point (8 h).

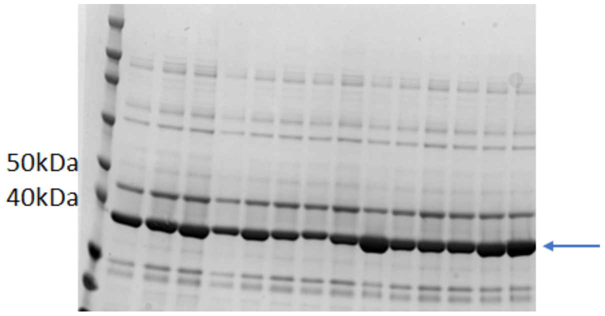

Figure S4. SDS-PAGE analysis of purified recombinant hOTC variants. All 87 hOTC variants were analyzed by SDS-PAGE to establish purity and confirm protein concentrations as determined by A280. Shown here are 14 of the 87 variants. The blue arrow indicates OTC. All proteins were determined to be >80% pure. Blot represents the full-length image with no modifications.

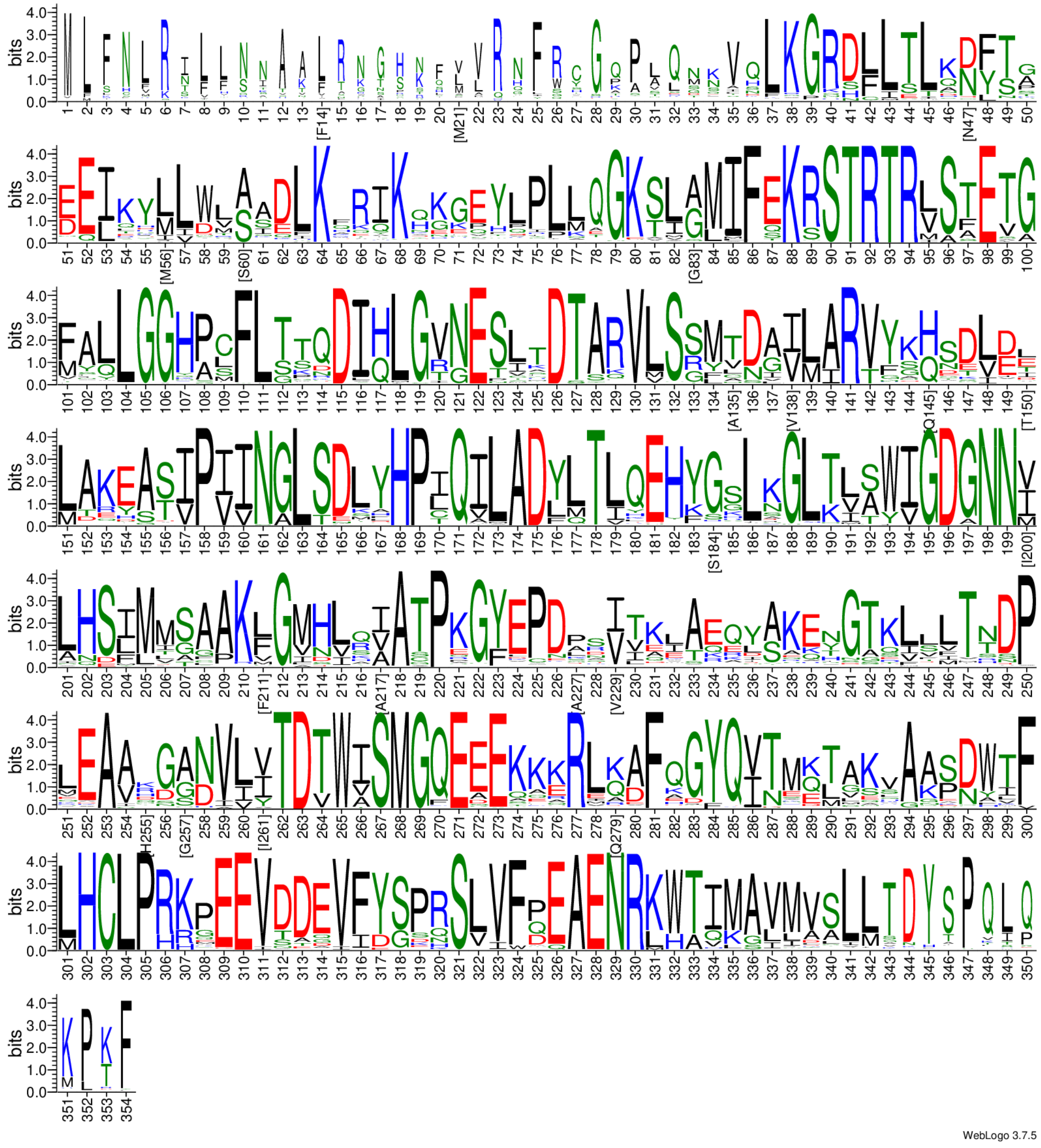

Figure S5. Weblogo of top 500 BLAST hits for human OTC (UniProt P00480) from the ‘nr’ database. The sequences were aligned by MAFFT[1].

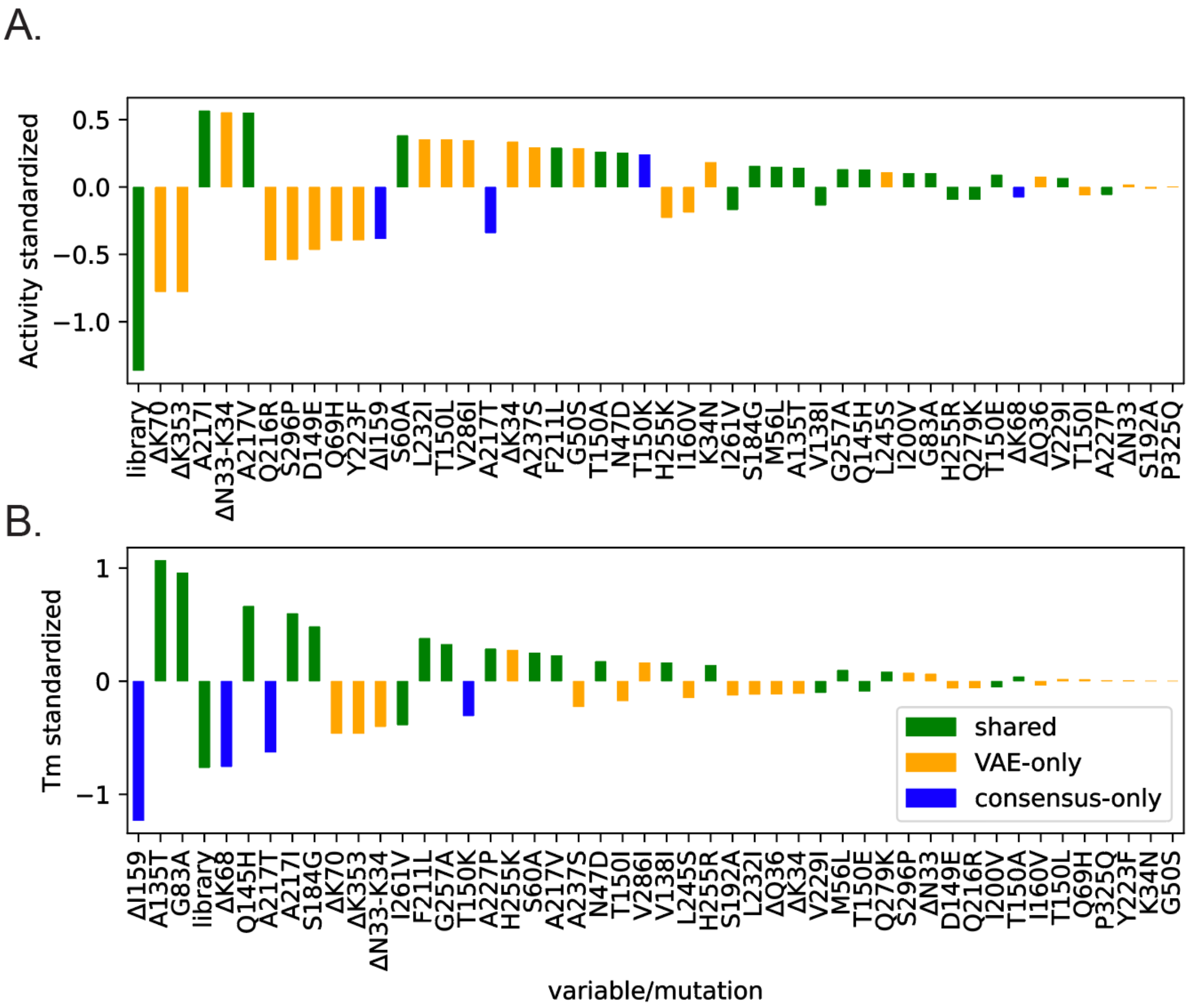

Figure S6. Coefficients for (A) specific activity and (B) T_m after regression with Ridge regularization.

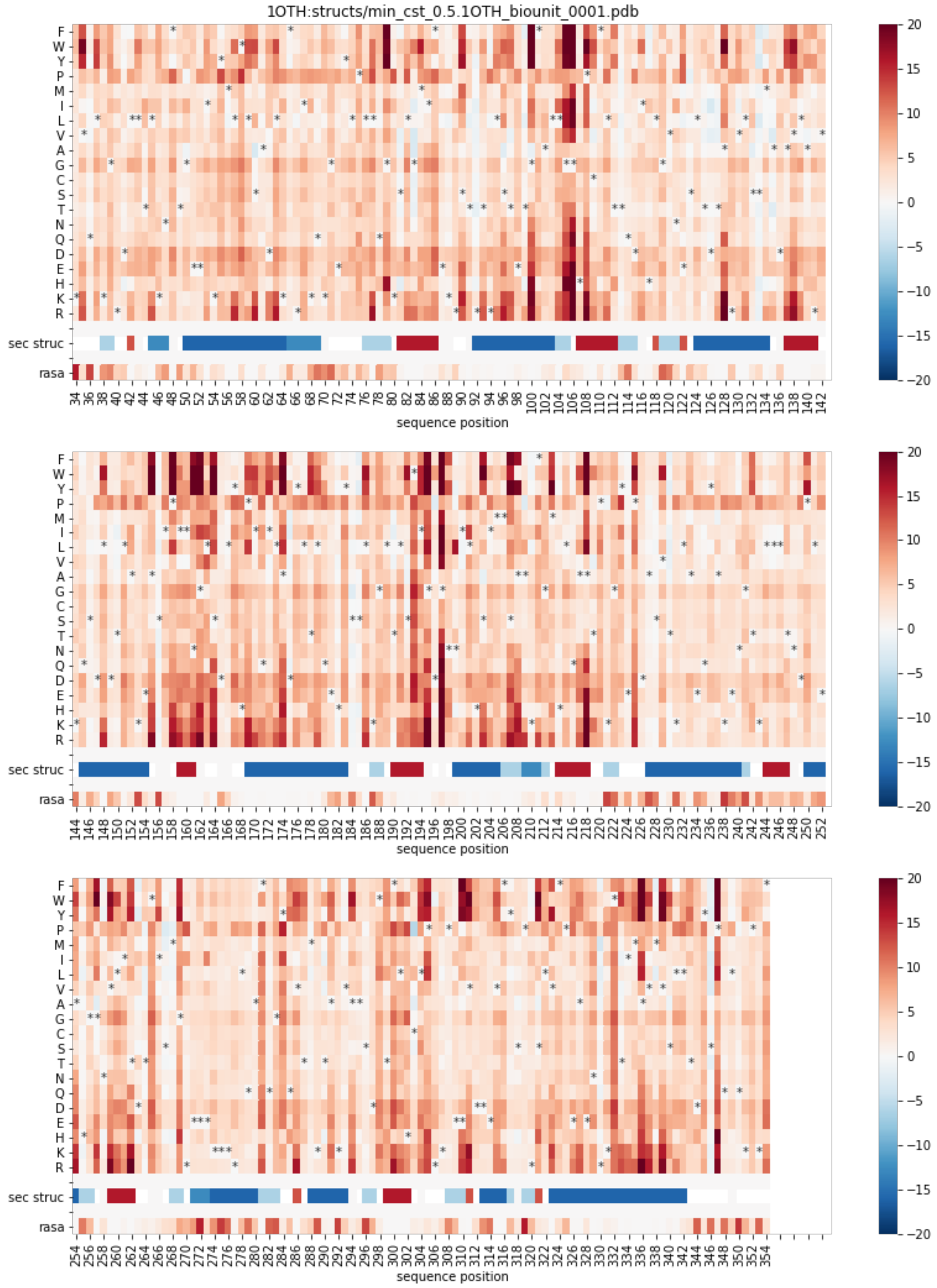

Figure S7. ΔΔG heatmap of all possible single substitutions using the Rosetta ddg_monomer protocol[2], based on a high-resolution crystal structure of hOTC bound to a bisubstrate analog (PDB: 1OTH). "rasa" is relative solvent accessible surface area: solvent accessible surface area (SASA) normalized by the SASA for an idealized tripeptide (Ala-Xaa-Ala), where Xaa is the amino acid of interest (in this case, the wildtype amino acid at that position).

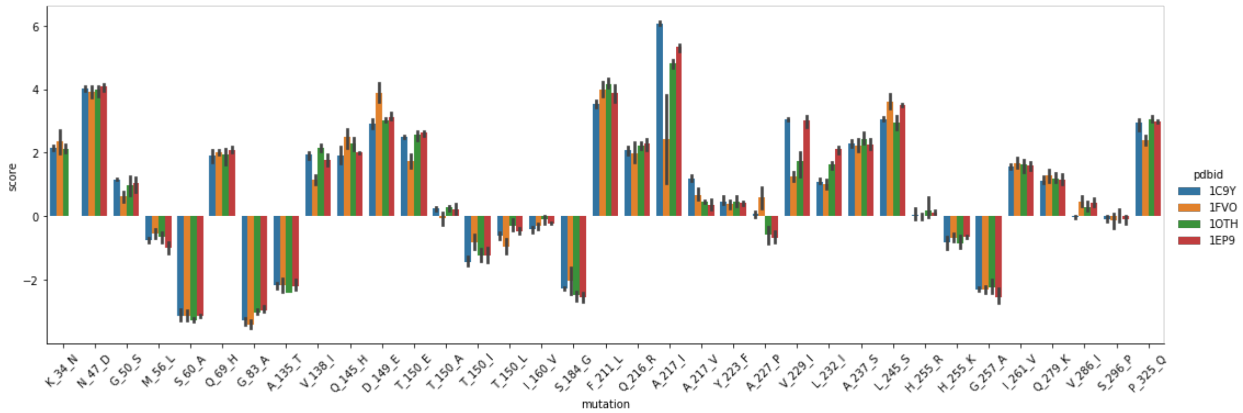

Figure S8. ΔΔG calculations for VAE-specific substitutions.

References

1. Katoh K, Misawa K, Kuma K, Miyata T. MAFFT: a novel method for rapid multiple sequence alignment based on fast Fourier transform. *Nucleic Acids Res*. 2002;30(14):3059-3066. doi:10.1093/nar/gkf436

2. Kellogg EH, Leaver-Fay A, Baker D. Role of conformational sampling in computing mutation-induced changes in protein structure and stability. *Proteins: Structure, Function, and Bioinformatics*. 2011;79(3). doi:10.1002/prot.22921
